# Green Analytical Workflows: Recent Advances in Sample Preparation and Instrumental Analysis of Food Contact Materials

**DOI:** 10.3390/molecules31173063

**Published:** 2026-08-31

**Authors:** Lorenzo Cortesi, Nicolò Riboni, Clarissa Facchini, Federica Bianchi

**Affiliations:** 1 Department of Chemistry, Life Sciences and Environmental Sustainability, University of Parma, Parco Area delle Scienze 17/A, 43124 Parma, Italy; lorenzo.cortesi@unipr.it (L.C.); clarissa.facchini@unipr.it (C.F.); 2 CIPACK (Interdepartmental Center for the Packaging), University of Parma, Parco Area delle Scienze 181/A, 43124 Parma, Italy

**Keywords:** packaging, IAS and NIAS, green metrics, mass spectrometry, extractable and leachable substances

## Abstract

Food contact materials (FCMs) represent a major route of exposure to chemicals since they can migrate from packaging into food, raising health concerns. Due to its heterogeneous nature and complexity, the monitoring of chemicals present in or released by FCMs poses a significant analytical challenge, requiring both target methods for the quantitative determination of both intentionally and non-intentionally added substances (IAS and NIAS) and untargeted approaches to fingerprint the material and identify previously unknown IAS and NIAS. In recent years, green analytical chemistry has become fundamental for method development, promoting safer, faster and more sustainable workflows with reduced solvent consumption, waste generation and environmental impact. This review discusses recent advances in green analytical workflows for FCM characterization, with emphasis on sample preparation and instrumental analysis, covering the state of the art from 2020 to 2026.

## 1. Introduction

Food contact materials (FCMs) and articles are materials and articles intended to come into contact with food, already in contact with food, or reasonably be expected to come into contact with food or transfer their constituents to food under normal or foreseeable conditions of use [1]. FCMs comprise a heterogeneous class of materials, including plastics, paper, glass, adhesives, printing inks, multilayer structures, and emerging bio-based, biodegradable, and recycled materials. Due to their direct contact with foodstuffs, these materials represent one of the most relevant routes of chemical exposure for consumers, since low-molecular-weight substances can migrate from packaging into food, thus raising concerns about food safety and potentially altering the organoleptic properties of food products [2,3,4]. Migration tests are therefore required to demonstrate compliance with current regulations [5,6,7] and to investigate the transfer of chemical substances from FCMs to food or food simulants under specific time–temperature conditions. In this context, the analytical characterization of migrating compounds is a challenging task because FCMs contain both intentionally added substances (IAS), including monomers, plasticizers, antioxidants, stabilizers, processing aids, photoinitiators, lubricants and pigments, and non-intentionally added substances (NIAS), deriving from impurities, side reactions, degradation processes, recycling, contaminants, adhesives, inks and residues from the manufacturing chain [2,3,8,9].

Although migration testing is essential for assessing FCMs safety, it cannot provide a comprehensive view of the compounds present within the material. Therefore, complementary approaches based on extraction or direct analysis of the packaging material are required to obtain a broader chemical characterization of IAS and NIAS. Exhaustive or accelerated extraction procedures are commonly applied to analyze the extractable substances [2,3,8,10]. The selection of the extraction/pretreatment strategy depends on both the physiochemical properties of the analytes and the nature of the FCM. In this context, headspace extraction (HS) [11,12,13], purge-and-trap (P&T) [14,15], and thermal desorption (TD) [16] are applied for the extraction of volatile and semi-volatile compounds, whereas liquid extraction [17], ultrasound-assisted solvent extraction (UAE) [18,19] solid-phase extraction (SPE) [20], and dissolution/precipitation [21] are suitable for semi-volatile and non-volatile substances. Direct analysis of the packaging material by ambient mass spectrometry (AMS) or Matrix-Assisted Laser Desorption/Ionization (MALDI)-MS can further reduce sample handling and provide rapid screening information, although confirmatory chromatographic methods are often required for reliable identification and quantification [22,23].

While FCM analysis aims to determine IAS and NIAS, most green analytical approaches are based on targeted analysis or suspect screening. Targeted methods are generally developed for the quantitation of known classes of specific migrants, including plasticizers, antioxidants, photoinitiators, bisphenols, oligomers and flame retardants. These approaches are particularly important for regulatory compliance and must be performed in accordance with the requirements and analytical conditions prescribed by the applicable legislation. In contrast, non-targeted analysis aims to detect the broadest possible range of substances present in a sample. The main challenge is related to the very different chemical natures of compounds that may originate from impurities, degradation, polymerization by-products, contamination or interactions among different packaging components [2]. This chemical complexity, together with the wide range of physicochemical properties and concentration levels involved, makes the comprehensive detection and characterization of NIAS particularly challenging. The identification of detected compounds requires cutting-edge instrumentation, appropriate data-acquisition strategies, feature prioritization, updated and dedicated databases, reliable spectral-library matching protocols, and comprehensive data interpretation. In this context, chemometrics plays a fundamental role in reducing data complexity, clustering samples, identifying discriminant features, and prioritizing compounds potentially related to material composition, processing conditions, recycling or degradation processes, and migration behavior. The confidence level of compound identification should be reported according to harmonized protocols, such as the Schymanski scale [24], while the toxicity of annotated compounds can be evaluated using available data or through structure-based approaches such as the Cramer rule [25]. One of the major limitations of non-targeted strategies is related to the lack of harmonized procedures, leading to variability in data acquisition, feature extraction, prioritization criteria, database searching, and reporting of identification confidence. Consequently, the comparison of NIAS profiles across different studies and laboratories remains challenging, limiting data comparability, reproducibility, and the broader implementation of non-targeted approaches in FCM safety assessment [2]. In this context, the development of harmonized non-targeted workflows is crucial to ensure robust and comparable results while minimizing resource consumption and unnecessary analytical efforts.

In recent years, mass spectrometry-based techniques have emerged as key analytical platforms for FCM characterization, providing high sensitivity, selectivity and structural information [2,3]. Gas chromatography-mass spectrometry (GC-MS), liquid chromatography-mass spectrometry (LC-MS), and AMS have been successfully applied for targeted and untargeted analysis of IAS and NIAS from extracts, migration solutions and directly from the material. Recent advances in high-resolution mass spectrometry (HRMS) and ion mobility spectrometry (IMS) have further expanded the possibilities of targeted and untargeted workflows, enabling accurate mass measurements, fragmentation-based structural elucidation through tandem mass spectrometry analysis (MS/MS), and the separation of isomeric or isobaric species through collision cross-section values. These features are fundamental for the untargeted investigation of IAS and NIAS, whose identification is often complicated by the absence of reference standards, limited spectral databases, and the presence of structurally related oligomers [2,3,4].

The development of analytical strategies able to detect and quantify IAS and NIAS in extracts, food simulants or real matrices requires appropriate sample preparation, preconcentration and clean-up procedures. These steps are pivotal for ensuring efficient analyte extraction and enrichment while minimizing contamination, degradation and analyte loss. In this context, the development of sustainable analytical solutions for assessing FCM safety is gaining increasing interest. Green analytical chemistry (GAC) aims to reduce the environmental and health impact of analytical procedures while maintaining the quality of analytical information [26]. An analytical method can be considered green when it reduces the amount and toxicity of solvents and reagents, waste generation, energy consumption, analysis time, and operational steps while improving operator safety, without compromising analytical performance in terms of sensitivity, selectivity, recovery, precision, and identification confidence. Method greenness should be assessed by considering the overall analytical workflow rather than individual steps. Therefore, sample preparation procedure, the number of procedural steps, operational energy consumption, analytical throughput, and overall method performance should all be taken into account when evaluating the environmental sustainability of an analytical approach. This concept is especially relevant for FCM analysis, where sample preparation and chromatographic separation may involve significant amounts of organic solvents, long extraction times, extensive sample handling and energy-consuming instrumentation. The greenness of FCM analytical workflows can be improved by considering both sample pretreatment and instrumental analysis [27].

In the context of sample pretreatment, the use of renewable, sustainable and bio-based solvents have been proposed together with the development of solvent-free, miniaturized, automatized and high-throughput procedures [26,27,28,29]. Green sample preparations redesign conventional extraction approaches according to sustainability criteria, while maintaining extraction, enrichment, and clean-up performance [26,30]. In this scenario, headspace extraction, green solvent extraction, solid-phase microextraction (SPME), dispersive solid-phase extraction (DSPE), magnetic solid-phase extraction (MSPE), liquid–liquid-microextraction (LLME), dispersive liquid–liquid microextraction (DLLME), and deep eutectic solvent (DES)-based extraction have been proposed for the extraction of compounds from FCMs. In addition, novel green materials have been designed to improve targeted extraction of leachable toxic compounds from FCM extracts, food simulants and real food samples.

However, GAC is not limited to sample pretreatment; it also extends to instrumental analysis, which is often associated with high solvent consumption, the use of toxic solvents, and significant waste production. In this regard, supercritical fluid chromatography (SFC) represents an attractive separation technique for GAC [27,31]. Moreover, direct analysis by MALDI-MS and AMS has emerged as a green approach, enabling the analysis of samples in their native state with minimal or no sample preparation [22,32]. Direct analysis in real time (DART), atmospheric solids analysis probe (ASAP), and MALDI-MS have been proposed for the rapid screening of oligomers and other substances from FCMs.

This review provides an overview of the most relevant green analytical strategies applied to the analysis of FCMs, with particular emphasis on their role in the detection of IAS and NIAS, in the 2020–2026 period. The literature search was performed using SciFinder, ScienceDirect (Elsevier), ISI Web of Science, Scopus and Google Scholar. Publications from January 2020 to July 2026 were considered. The search was carried out using combinations of the following keywords: green analytical chemistry, food contact materials, plastics, packaging analysis, intentionally added substances, non-intentionally added substances, mass spectrometry, green sample preparation, green solvent extraction, supercritical fluid chromatography, matrix-assisted laser desorption/ionization mass spectrometry, ambient mass spectrometry, direct analysis in real time, solid-phase microextraction, dispersive solid-phase extraction, liquid–liquid microextraction, and deep eutectic solvents.

The first part of this review will focus on green and miniaturized sample pretreatment approaches, including headspace-based techniques, green solvent extraction, SPME, DSPE, MSPE, LLME, DLLME, and the use of DESs. The second part will discuss greener instrumental strategies, focusing on SFC and direct/ambient mass spectrometry. An overview of the metrics used to assess the greenness of analytical methods will be provided in the final section of this manuscript. The aim is to highlight the transition toward the development of safer, faster and more sustainable analytical workflows for FCM characterization while providing enhanced sensitivity, selectivity and identification confidence.

## 2. Green Sample Pretreatment

Sample pretreatment plays a central role in FCM analysis, as the extraction strategy directly affects analyte recovery, enrichment, and matrix clean-up. However, no single extraction technique can provide exhaustive coverage of the chemical space of substances released from FCMs; therefore, various green approaches have been proposed. These strategies are selected according to the volatility, polarity and expected concentration of the analytes, as well as the nature of the material, the food simulant or the extract under investigation. Overall, a green sample pretreatment strategy for FCM analysis aims to minimize the amount and toxicity of solvents and reagents, reduce waste generation, energy consumption and extraction time, and limit sample handling through simplified, miniaturized or automated workflows [33]. At the same time, pretreatment must provide adequate extraction efficiency, enrichment capability and matrix clean-up to guarantee the reliability of the analytical method.

### 2.1. Headspace Extraction

Headspace-based techniques represent one of the most relevant green sample pretreatment strategies for the analysis of volatile and semi-volatile compounds released from FCMs. These approaches exploit the partitioning of analytes between the material, the liquid phase or simulant, and the gas phase, enabling solvent-free extraction with limited sample handling. However, their applicability is limited to volatile and semi-volatile compounds, and their efficiency strongly depends on the analyte volatility, matrix composition, extraction temperature and time, sorbent chemistry, and desorption conditions [3]. P&T and dynamic headspace (DHS) are the most applied HS techniques for FCMs analysis since they improve the extraction capability compared with equilibrium-based methods. Hosono et al. developed a targeted P&T-GC–MS method for the determination of 21 representative flavor compounds sorbed by plastic packaging materials, including polyethylene terephthalate (PET), polyethylene (PE), and polypropylene (PP) films and multilayer pouches [14]. Compared with a conventional solvent extraction approach based on diethyl ether, the developed workflow reduced the amount of sample (a few cm^2^ vs. 100–200 cm^2^ of material) and time (1 h vs. 48 h) while avoiding the use of organic solvents. The method was characterized by limits of detection (LODs) at the ng level, good precision (relative standard deviations, RSDs < 10%), and trueness (95–105%).

Vázquez-Loureiro et al. proposed an untargeted workflow based on P&T-GC-MS for the identification of volatile compounds in bio-based and biodegradable FCMs [15]. In addition, a GC-HRMS approach was applied for the analysis of semi-volatile compounds after acetonitrile extraction. Therefore, while the P&T approach suits the GAC principles, the acetonitrile extraction used for semi-volatiles represents a complementary but less green solvent-based approach. Under optimal extraction conditions, 125 volatile compounds were detected in bio-based and/or biodegradable food contact materials, of which 100 were tentatively identified. These included monomers, starting substances, authorized additives, solvent residues, and reaction and degradation products.

Concha-Graña et al. developed a targeted in-tube DHS-GC-MS/MS method for the quantitative analysis of 47 additives in plastic samples [11]. The analytes comprised phthalic acid esters (PAEs), bisphenols, adipates, citrates, benzophenones, organophosphorus compounds and other classes of additives. The extraction was based on multiple aspiration of the headspace above the sample using a gas-tight syringe through a sorbent-filled tube, allowing analyte enrichment before thermal desorption into the GC system. The method was tested for the analysis of different polymeric materials, and matrix-matched calibration with labelled surrogate standards was used to minimize matrix effects. LODs were in the 0.0002–4.8 µg/g range, precision showed RSDs < 26%, while recoveries in the 70–128% range were obtained for the 47 target additives.

Finally, Koster et al. performed an interlaboratory study to evaluate the performance of HS-GC-MS, GC coupled with flame ionization detection (GC-FID), and LC-HRMS for the identification and quantitation of NIAS [13]. In the study, 13 laboratories applied their in-house methods to analyze plastic laminates based on HS extraction and migration using Tenax^®^ and 50% *v*/*v* ethanol. The study demonstrated significant inconsistencies across laboratories, mainly due to differences in analytical techniques, selected standards, libraries and reporting practices. Improved consistency was observed when aligned methodologies were used, thus emphasizing the need for harmonized procedures for the analysis of NIAS to ensure comparable results, particularly at low concentration levels.

### 2.2. Green Solvent Extraction

Green solvent extraction has been proposed as a promising strategy to replace toxic conventional organic solvents with more sustainable alternatives for the characterization of extractable compounds from FCMs [28,29]. Unlike migration testing, which is primarily designed to assess the transfer of substances under defined conditions and demonstrate compliance with regulatory requirements, solvent extraction can provide complementary information on a broader range of extractable or potentially detectable compounds, including substances that may not migrate under standard compliance conditions. Therefore, green solvents represent a valuable approach for screening, fingerprinting and comprehensive characterization of FCMs while contributing to the reduction of the environmental impact associated with sample preparation. However, the definition of a green solvent should not be limited to its origin or the replacement of conventional hazardous solvents. It should also consider the overall environmental, health and safety profile of the solvent, including toxicity, biodegradability, consumption, production energy demand, end-of-life recovery or disposal, physicochemical suitability, cost and practical compatibility with the analytical workflow. In this perspective, bio-based or biomass-derived solvents cannot be automatically considered greener than petrochemical solvents [34]. Recent solvent-selection guides specifically designed for analytical chemistry, like the Green Solvent Selection Guide, provide structured and evidence-based tools to promote the selection and adoption of greener and safer solvent alternatives [35]. Accelerated extraction strategies, including UAE, microwave-assisted extraction (MAE), or pressurized liquid extraction (PLE), have been applied to reduce solvent toxicity, solvent volume, extraction time and sample handling while increasing recoveries.

Hernández-Fernández et al. evaluated limonene, dichloromethane and cyclohexane as extraction solvents in combination with Soxhlet extraction, UAE and MAE for the targeted determination of the slip additive cis-1,3-docosenamide (erucamide) and nine oxidative degradation by-products in polypropylene samples by GC-MS [36]. MAE provided the highest erucamide recovery, exceeding 96%; UAE was also characterized by promising extraction performance, with a recovery of 94%. By contrast, Soxhlet required prolonged extraction and promoted the degradation of the target compound, with the formation of different degradation by-products. A similar approach was applied by the same group for the quantification of Irgafos P-168 and its degradation products in PP/PE composite materials [37], confirming the previous results. These findings support the use of limonene as a greener alternative for UAE and MAE extraction of hydrophobic additives from polyolefin matrices.

Green solvent extraction has also been explored in the context of recycled food-contact polyolefins. Blázquez-Martín et al. developed a PLE-GC/MS method for the determination of seven model contaminants in post-consumer and recycled high-density polyethylene (HDPE), namely chloroform, toluene, chlorobenzene, methyl salicylate, phenyl cyclohexane, benzophenone and methyl stearate [38]. The analytical extraction was performed by PLE using acetone, but it required three extraction cycles. Good analytical performance was obtained, with LODs below 0.5 µg/L for most contaminants, recoveries in the 97–106% range (except for chloroform, at 71%), and good repeatability and intermediate precision, generally below 11% and 17%, respectively. The method was then applied to evaluate a novel green recycling strategy based on the dissolution/precipitation of HDPE using limonene. The recycling method was shown to have high cleaning efficiency, proving its potential in the recycling of HDPE.

Very recently, cyclopentyl methyl ether (CPME) was tested as a green solvent for the untargeted characterization of volatile and semi-volatile compounds from bio-based FCMs [18,19]. In the first study, a UAE extraction using various conventional and green solvents was optimized to improve the sustainability of the analytical workflow for the characterization of bio-based tableware samples. Design-of-experiment and quality-by-design approaches were applied to optimize the extraction conditions while guaranteeing the reliability of results. CPME was characterized by the highest extraction performance, allowing the GC-MS detection of 39 volatile compounds belonging to different chemical classes and a correct classification of bio-based samples according to their polymeric composition. The same extraction strategy was then applied for the development of an untargeted workflow based on GC-Orbitrap-HRMS and GC×GC–MS, with the aim of improving the identification confidence and chemical coverage of IAS and NIAS. GC-Orbitrap-HRMS, combined with chemometrics, enabled the prioritization of discriminant features associated with material composition and maximum end-use temperature, while GC×GC–TOF-MS provided enhanced chromatographic separation and helped recognize structured chemical patterns. The approach allowed the detection and tentative identification of several material-related compounds, including lactic acid derivatives, linear and cyclic polyl (lactic) acid (PLA) oligomers, hydrocarbons, citric acid esters and mineral oil saturated hydrocarbons.

Supramolecular solvents (SUPRASs) have been also proposed for developing green extraction workflows. In particular, García-Cansino et al. developed a SUPRAS-based all-in-one extraction combined with LC-HRMS for suspect screening of food contact chemicals from plastic- and Tetra Brik-based FCMs [17]. This approach was specifically designed to overcome one of the main limitations of conventional solvent extraction, namely the strong dependence of the extracted chemical space on solvent polarity. Conventional FCM extraction often requires repeated solvent treatments with multiple solvents, followed by clean-up or evaporation/reconstitution steps, which are in contrast with the GAC principles. Conversely, SUPRAS extraction integrated food contact chemical (FCC) extraction and matrix clean-up in a single step, requiring only vortexing and centrifugation and only 100 µL of SUPRAS per sample. The selected SUPRAS, based on 1-decanol in ethanol–water media, behaved as a restricted-access material, improving interference removal and reproducibility. Using an in-house suspect list of 1389 chemicals, the method enabled the identification of 29 FCCs, including 9 IAS and 20 NIAS, belonging to eight chemical classes and covering a broad polarity range from logP −5.2 to 9.8.

Overall, these studies suggest that green solvents can represent viable alternatives to conventional solvents for the extraction of FCCs, especially when combined with MAE or UAE. However, solvent selection must be evaluated together with extraction efficiency, polymer swelling or dissolution, analyte stability, the formation of degradation artefacts, matrix effects, compatibility with GC- or LC-based platforms and the analytical aim of the study. Therefore, extraction conditions have to be optimized on case-by-case basis according to the material, analyte class and screening strategy.

### 2.3. Solid-Phase Microextraction

SPME is one of the most widely applied green sample pretreatment techniques for the analysis of volatile and semi-volatile compounds from food contact materials (FCMs), packaging extracts, food simulants and packaged food samples [3]. Its main advantages are related to the integration of sampling, extraction and preconcentration in a single step, with limited or no solvent consumption. SPME can be applied in HS mode for the extraction of volatile compounds directly released from packaging materials, food simulants or extracting solutions or in direct immersion (DI) mode. One of the main factors influencing the extraction capability of SPME is the nature of the coating material: while commercial coatings are characterized by low selectivity and limited extraction capacity, novel nanostructured and porous materials have been proposed to increase both extraction selectivity and efficiency [39]. In the context of FCMs, SPME extraction has been applied for both targeted and untargeted analysis.

#### 2.3.1. Targeted Approaches Using Commercial SPME Coatings

Targeted SPME methods using commercial fibers have been mainly applied to plasticizers, siloxanes, and mineral oil aromatic hydrocarbons (MOAH) from plastic materials and laminates (Table 1).

Alshehri et al. developed a DI-SPME-GC-MS/MS method for the quantitation of six PAEs, namely dimethyl phthalate (DMP), diethyl phthalate (DEP), diisopropyl phthalate, diisobutyl phthalate, di-n-butyl phthalate, and di-ethylhexyl phthalate (DEHP), in PET-bottled water [40]. Similarly, De Vietro et al. proposed a targeted DI-SPME-GC/MS method for assessing the migration of DMP, DEP, dipentyl phthalate (DPP) and DBP from plasticizer-free food packaging polymers into food simulant media [41]. The approach was applied to investigate the occurrence of PAEs as NIAS in aqueous, alcoholic and fatty-food simulants exposed to materials labelled plasticizer-free, detecting the presence of PAEs for all materials in at least one food simulant.

Asensio et al. developed a HS-SPME-GC-MS method to identify and quantify volatile silicone oligomers and other potential migrants released from silicone molds using Tenax^®^ as a dry food simulant [42]. Both DVB/CAR/PDMS and PDMS fibers were evaluated, with the latter providing the best extraction performance due to the different polarity of the compounds investigated. The HS-SPME-GC-MS analysis resulted in the identification of 47 compounds in Tenax ethanolic extracts^®^, including 12 cyclic and 1 linear siloxane and 34 other volatile migrants, including naphthalene, 1,1-biphenyl, BHT, alkanes, fatty acid esters, benzophenone, and dodecyl acrylate.

Jaén et al. proposed a HS-SPME-GC-MS method for the identification of MOAH from hot-melt adhesives used in multilayer food packaging [43]. This approach was designed to provide rapid and direct detection of volatile MOAH markers from solid adhesive samples before investigating the migration of the MOAH fraction into Tenax^®^, using a validated protocol based on organic solvent extraction and GC-FID analysis. Sixteen MOAH markers were investigated, of which twelve were identified in adhesives and eight were detected after migration, with values ranging from 0.62 to 21.33 µg/dm^2^. The method was particularly effective for the more volatile MOAH markers, whereas heavier MOAH components required conventional solvent-based extraction followed by GC-FID analysis.

Guan et al. developed a DI-SPME-GC-MS method for the determination of substances migrating from plastic multilayer pouches and cartons into food simulant D1 [44]. Polar and apolar commercial fibers were tested, with 65 μm PDMS/DVB as the highest extraction performance. Forty-two compounds were detected, of which 36 were successfully identified, including hydrocarbons, plasticizers, slip agents, lubricants, antioxidants, degradation products, solvents and initiator residues. A risk assessment evaluation was also performed, highlighting the risk of the potential migration of toxic substances from multilayer packaging materials into milk.

Overall, commercially available SPME coatings represent a suitable green alternative for targeted analysis, owing to their solvent-free operation, minimal sample handling, and low waste generation, although their limited selectivity may restrict extraction efficiency for specific analytes or chemically diverse target classes.

#### 2.3.2. Targeted Approaches Using Innovative SPME Coatings

To overcome the limited selectivity of commercial fibers, novel SPME coatings have been proposed for the extraction of FCCs (Table 1).

Owing to their widespread use and toxicity, PAEs are considered priority contaminants in FCMs; therefore, considerable efforts have been devoted to improving their detection. Erdem et al. developed a biopolymer-ionic liquid-modified clay as a bionanocomposite coating for the DI-SPME-GC-MS analysis of six PAEs [45]. The coating was synthetized by intercalating montmorillonite clay with carboxymethyl cellulose and dicationic imidazolium-based ionic liquid. The novel coating outperformed the individual components, achieving LODs at the ng/L level and a lifetime exceeding 120 cycles.

Li et al. synthesized three hypercrosslinked polymers through Friedel–Crafts alkylation reaction between tetraphenylethylene and 1,4-bis (chloromethyl)benzene, 4,4′-bis (chloromethyl)-1,1′-biphenyl, and cyanuric chloride. The coatings were tested for the DI-SPME-GC-MS quantitative analysis of eight PAEs in environmental and bottled water [46]. The tetraphenylethylene-1,4-bis (chloromethyl)benzene coating provided the best extraction efficiency, with enrichment factors (EFs) in the 1634–21,909 range. Similarly, Xiang et al. developed a quinoxaline-based conjugated microporous polymer coating for nine PAEs in bottled liquid food samples using GC-MS [47]. The good extraction efficiency was ascribed to the large surface area and the hydrophobic and conjugated planar structure of the coating, which favored π–π and hydrophobic interaction retention with PAEs.

Metal–organic frameworks (MOFs) and covalent–organic frameworks (COFs) have been proposed as nanostructured and tunable alternatives to commercial fibers [39]. Rascón et al. proposed CIM-80 (Al) as a DI-SPME fiber coating for the determination of 14 PAEs in bottled water samples by combining DI-SPME extraction with ultra-high performance liquid chromatography (UHPLC)-MS/MS detection [48]. The coating was synthetized using a sustainable process; despite being only 3.5 µm thick, the coating exhibited performance statistically comparable to that of a commercial 50/30 μm PDMS/DVB/CAR fiber. Yuan et al. developed a stainless-steel fiber with a gully-like surface obtained by electrochemical etching and coated with a COF based on 1,3,5-tris (aminophenyl) benzene and 2,5-dimethoxyterephthalaldehyde [49]. The novel coating was tested for the DI-SPME-GC-FID determination of five PAEs in bottled tea beverages, obtaining EFs in the 268–2657 range for DEHP and DPP while maintaining stable performance for at least 180 cycles.

Li et al. developed an oxygenated carbon nanotube cage-coated SPME fiber for the selective extraction of migrated aromatic amines from FCMs [50]. The material was obtained from ZIF-67 followed by calcination and oxidation; the coating was synthesized by chemical bonding between the oxidized carbon nanotube cages and a polydopamine layer, obtaining matched pore size, graphitic domains and abundant oxygen-containing groups. High selectivity toward aromatic amines over other aromatic compounds potentially migrating from FCMs was demonstrated. Moreover, the coating maintained stable performance for 150 extraction cycles, providing excellent reusability, in line with the principle of GAC.

Finally, Niu et al. proposed a dynamic vapor sorption strategy to rationally screen MOF materials for SPME coatings, targeting vinyl acetate, a monomer relevant to FCMs [51]. In this study, 13 MOFs were first screened by dynamic vapor sorption (DVS) using vinyl acetate as the target vapor, allowing the affinity of each material toward the analyte to be predicted before fiber fabrication. The DVS ranking was fully consistent with the extraction performance of the corresponding MOF/polyimide SPME probes. ZIF-68 was selected as the optimal coating, and the HS-SPME-GC-FID method was successfully validated, achieving a LOD of 0.1 ng/mL. These results demonstrated both the predictive value of DVS for coating selection and the applicability of the method to the specific migration testing of vinyl acetate.

As a final comment, it can be stated that the development of tailored non-commercial SPME coatings can improve extraction selectivity and sensitivity toward specific target analytes while preserving the intrinsic GAC advantages of SPME.

#### 2.3.3. Non-Targeted SPME Approaches

Besides targeted applications, SPME has also been increasingly used for the non-target screening of volatile and semi-volatile IAS/NIAS released from FCMs (Table 2). Su et al. optimized a DI-SPME-GC-MS method for the screening of migrants into simulants A, B and D2 substitutes, using response surface methodology [52]. The method extraction capabilities were evaluated in terms of detection limits, considering the signal/noise ratio, for 35 FCCs as model compounds, achieving LODs in the 0.1–14.1, 0.1–20.2 and 0.7–163.7 µg/kg range for food simulants A, B and D2, respectively.

SPME has also been coupled with olfactometry (HS-SPME-GC-O-MS) to investigate off-odors in recycled materials intended for food packaging by Paiva et al. [53]. The method was applied to recycled polypropylene subjected to forced contamination and recycling. Among the forty-five volatile compounds that were identified, nine showed odor activity. The use of a non-targeted approach allowed the identification of DEP, proposed as an IAS, while glycerin was identified as a NIAS associated with the production process. The results showed that contamination and recycling increased PP degradation and intensified off-odor formation. A similar HS-SPME-GC-O-MS strategy was applied by the same group to analyze recycled poly(lactic) acid (PLA) as FCM [54]. PLA pellets were subjected to a post-consumer contamination protocol, washing and mechanical recycling. The use of an untargeted workflow proved that the recycling cycle is able to promote NIAS formation, mainly through polymer chain degradation (accounting for 56% of the total), followed by NIAS generation during the recycling process (26.7%), while 20% of the detected NIAS could not be identified in databases (20%). Among the 34 compounds identified in the PLA samples, 14 compounds were characterized by distinct odor characteristics.

Wongphan et al. applied HS-SPME-GC-MS and UHPLC-HRMS for the screening and relative quantification of compounds migrating from a novel biodegradable food packaging material based on cassava thermoplastic starch and polybutylene adipate terephthalate [55]. The complementarity between SPME-GC-MS for the analysis of volatile migrants and HRMS-based LC methods for the determination of oligomeric and non-volatile compounds allowed the detection of fifteen volatile and semivolatile compounds, mostly deriving from polymerization or degradation processes, as well as the identification of cyclic PBAT oligomers. A comprehensive approach was also followed by Estremera et al., who proposed DI-SPME-GC-MS together with UHPLC-IMS-HRMS to investigate migrants released from vacuum cooking bags in three food simulants [56]. DI-SPME-GC-MS enabled the identification of 64 volatile compounds, with 64% classified as IAS and 36% as NIAS. Within the NIAS class, 46% of the compounds were labelled as contaminants, while 29% and 21% were classified as degradation products of additives and polymer, respectively. Finally, twenty-nine compounds were identified by UHPLC-IMS-QTOF. The same research group tested the use of commercial SPME Arrows for the HS-SPME-GC-MS characterization of recycled PET and polyolefins [57], analyzing both solid materials and migration extracts. Compared with conventional SPME fibers, SPME Arrows provided a larger sorptive-phase volume and improved mechanical robustness, which enhanced extraction capacity, sensitivity and reproducibility. Under the optimized conditions, 121 and 162 compounds were identified in 10% *v*/*v* ethanol and 3% *w*/*v* acetic acid extracts of rPET, respectively, while 178 compounds were detected in the extracts of recycled polyolefins. Finally, a 15-analyte validation mixture demonstrated that SPME Arrow was able to improve sensitivity, achieving responses 7–14% higher than conventional SPME.

Finally, Murat et al. tested the use of microchamber-based SPME and pyrolyzer/thermal desorption coupled to GC-MS for solvent-free screening of potential extractables and leachables from packaging materials [16]. Seven cosmetic plastic packaging samples made of PP, PE and styrene-acrylonitrile copolymer were tested, identifying 35 volatile and semi-volatile compounds, including phthalates, alkanes, styrene, cyanide derivatives, degradation products, impurities, additives, plasticizers and monomers. Among the 14 PAEs identified by the microchamber-SPME extraction, four were also quantified in food simulants, in the 12.4–491 μg/L range. The authors emphasized that these thermal/headspace techniques should be regarded as complementary to migration testing, since they enable the identification of potential leachable substances from the material before their subsequent quantitation.

Overall, the reviewed studies demonstrate that SPME is a versatile and environmentally friendly sample pretreatment strategy for FCM analysis. Targeted approaches using commercial coatings remain valuable for the routine monitoring of selected migrants, while the development of innovative coatings has improved selectivity, sensitivity and durability for specific classes of analytes. Non-targeted SPME workflows are particularly useful for volatile fingerprinting of FCMs and off-odor assessment. However, SPME mainly covers volatile and semi-volatile compounds, while low-volatile additives, oligomers and polar non-volatile NIAS require complementary LC-HRMS-based methodologies. Therefore, SPME should be regarded as a key green sample preparation technique within integrated multi-platform workflows aimed at comprehensive IAS/NIAS characterization. In the context of non-targeted analysis, the use of comprehensive analytical techniques, particularly HRMS, entails substantial energy consumption due to prolonged instrumental operation and intensive data processing. Since energy demand is an important factor in greenness assessment, it could decrease the overall environmental sustainability score of the analytical workflow.

### 2.4. Dispersive and Magnetic Solid-Phase Extraction

DSPE and MSPE have gained increasing interest as green sample pretreatment strategies for the extraction, clean-up and preconcentration of substances from FCMs. Unlike conventional SPE, DSPE and MSPE are based on the direct dispersion of the sorbent into the liquid sample or extract, which improves contact between the sorbent and analytes, accelerates mass transfer and reduces extraction time [58]. Using magnetic cores, the sorbent can be rapidly recovered using an external magnet, avoiding time-consuming centrifugation steps. From a GAC perspective, these approaches are attractive because they generally require small amounts of sorbent and reduced elution volumes. Different materials, including MOFs, COFs and carbon-based sorbents have been proposed for targeted analysis of compounds from FCMs (Table 3).

Lian et al. developed a DSPE-UHPLC-HRMS method for the extraction of bisphenol A (BPA) and bisphenol AF migrating from disposable plastic packaging materials [59]. The developed material was based on cetyltrimethylammonium bromide-intercalated zinc oxide (ZnO@CTAB): CTAB was used to modify the hydrophilic ZnO into a more hydrophobic nanomaterial, thus enhancing the interaction with bisphenols. The material was applied to test the migration from disposable plastic bags, enabling the detection of bisphenols and investigation of the influence of food simulant polarity on their migration behavior. Zhang et al. combined a dissolution–precipitation approach with MSPE by developing a sodium alginate/MOF-derived magnetic multistage porous carbon material, MIL-101 (Fe)/SA-CAs, for the selective analysis of antioxidants and UV stabilizers from PLA samples [21]. The hierarchical porosity of the MIL-101 (Fe)/SA-CAs sorbent, comprising micro-, meso- and microporous domains, facilitated the diffusion of target analytes through the larger pores while increasing the exposure of active adsorption sites through smaller pores, thereby promoting the rapid and efficient extraction of antioxidants and UV stabilizers (total extraction time 1 min).

Several DSPE methods have been applied to packaged foods as the final exposure matrices. Nabil and coworkers developed a DSPE-HPLC-UV method for the determination of phenolic antioxidants in milk samples packaged in PE [60]. Carbon-derived MIL-88 (Fe) was proposed as a sorbent, while vortexing (VA) was applied to accelerate extraction, followed by desorption using 125 μL choline chloride (ChCl):1,4-butanediol. The method was applied for the analysis of 25 milk samples, investigating the effect of storage time and temperature on the migration of phenolic antioxidants. Although the use of a DES as the eluent avoids the need for conventional toxic organic solvents during the desorption step, the procedure requires different pretreatment steps and extensive sample handling. Smiełowska et al. investigated the migration of alternative plasticizers (adipates, citrates, and cyclohexane dicarboxylates) from packaging into protein supplements and the associated human exposure [61]. Sample preparation for protein supplement samples combined UAE, DSPE and SPE clean-up, while the corresponding packaging materials were extracted by UAE using acetonitrile. Also, in this case, the combination of multiple extraction/clean-up processes can affect the evaluation of the overall greenness of the proposed workflow. Considering the lack of multi-analyte methods, Miserez et al. developed multi-analyte atmospheric-pressure GC-MS/MS and LC-MS/MS methods for the quantitation of 110 chemicals from printing inks and adhesives used in plastic FCMs [62]. Quick, Easy, Cheap, Effective, Rugged and Safe (QuEChERS) extraction was performed, and DSPE clean-up was performed using C18 or C18/PSA, depending on the matrix. The UHPLC-MS/MS and GC-MS/MS methods were validated for 59 and 51 compounds, respectively, considering five different food matrices, achieving LOQs below or close to the European regulatory limit of 10 µg/kg. Coupling QuEChERS with DSPE provided efficient clean-up and reduced matrix interferences while maintaining low solvent consumption and a relatively simple sample preparation workflow, thus improving the overall greenness and applicability of the analytical method.

Other studies have focused on the determination of plasticizers and additives in beverages stored in plastic packaging. Jiang et al. synthesized magnetic microspheres decorated with a nitrogen- and oxygen-co-doped carbon shell for the extraction of target PAEs and magnetic retrieving of adsorbed compounds [63]. The MSPE-UHPLC-UV method was applied for the analysis of PAEs in bottled water, representing a competitive and sustainable alternative for PAE determination; the workflow combined minimal sorbent consumption, short extraction time, satisfactory sensitivity, and compatibility with widely available UHPLC-UV instrumentation. Hosseini et al. proposed a ternary NiCoMn-MOF composite as a DSPE sorbent for DEP, DBP, DiPB and DEHP determination in fruit juice samples prior to GC-FID analysis [64]. Doping the MOF allowed the tuning of the material properties, resulting in a large specific surface area, high porosity, and enhanced adsorption capacity through π-π interactions, obtaining EFs in the 325–375 range. Finally, the DSPE-GC-FID method was applied to fruit juices, detecting PAE presence in all samples and investigating the effects of storage temperature and time.

Overall, DSPE and MSPE represent promising sample preparation strategies for targeted FCM-related analysis. The main strengths are operational simplicity, short extraction time, reduced sorbent amount, the implementation of engineered materials and the possibility of magnetic sorbent recovery. However, current applications remain predominantly targeted and often require multiple steps to handle complex matrices. Therefore, DSPE should be considered as a powerful green clean-up and enrichment technique rather than a stand-alone approach for the comprehensive untargeted characterization of IAS/NIAS in FCMs.

### 2.5. Liquid–Liquid Microextraction

LLME and its dispersive configurations represent miniaturized alternatives to conventional liquid–liquid extraction for the analysis of migrants from FCMs, food simulants and packaged food matrices (Table 4). These approaches are based on the partitioning of analytes between an aqueous or hydroalcoholic phase and a small volume of extraction solvent, often assisted by VA, ultrasound, salting-out, effervescence or in situ phase formation [65,66]. Compared with conventional approaches, LLME reduces solvent consumption, extraction time and sample volume while providing analyte enrichment before chromatographic determination.

Miralles et al. developed a VALLE approach coupled with GC-HRMS detection for the targeted determination of 60 migrant substances from plastic FCMs, including aldehydes, ketones, plasticizers, phenol derivatives, acrylates and methacrylates [67]. The extraction was carried out after migration testing performed using simulants A, B, C and D1 and *n*-hexane as the extraction solvent. Combination with GC-HRMS provided high selectivity and accurate-mass confirmation, enabling the multi-analyte determination of FCCs. Mahdavianpour et al. developed a DLLME-HPLC-UV method for the determination of BPA migration from different FCMs, including cans, paper boxes and glass jars [68], using 1-undecanol as the extracting solvent and acetone as the dispersing solvent. Similarly, Karsauliya et al. developed a DLLME-UHPLC-MS/MS method for the determination of seven bisphenols in surface water, tap water and packaged drinking water [69]. The optimized DLLME procedure involved extraction using acetone and carbon tetrachloride, sonication, centrifugation, evaporation and reconstitution in acetonitrile before UHPLC-MS/MS analysis. Caldeirão et al. and Pereira et. al. applied a DLLME-GC-MS/MS method for the quantitative analysis of six PAEs and bis (2-ethylhexyl) adipate (DEHA) in bottled herbal-based soft drinks [70] and in commercial beers, respectively [71]. The extraction was based on the use of *n*-hexane as the extracting solvent and ethanol as the dispersive solvent. A VA-UAE-DLLME-GC-MS method was developed by Ianiri et al. for the determination of seven PAEs in hot drinks dispensed by vending machines [72]. Under optimal conditions, 250 µL of *n*-heptane was used for extracting 10 mL of sample, using UAE and VA for increasing the contact surface. The proposed approach was specifically designed to investigate PAE contamination in hot beverages under realistic vending conditions, accounting for the short-term effects of dispensing time and temperature and providing insight into the contamination potentially arising from both disposable cups and the plastic components of vending machines. Wang et al. combined salting-out-assisted LLE with DLLME for the determination of BPA and six analogues in canned coffee drinks by UHPLC-MS/MS [73]. The two-step strategy was based on LLE using acetonitrile and ammonium sulfate, followed by DLLME in water, using the previous phase as the dispersive solvent and dichloromethane as the extracting solvent. Although these studies demonstrated the sensitivity and simplicity of the DLLME procedure while reducing solvent consumption, limited greenness was achieved, since they still relied on conventional toxic solvents for the extraction procedure, namely *n*-hexane [67,70,71], *n*-heptane [72], dichloromethane [73], carbon tetrachloride [69], acetone [68,69], and acetonitrile [73]. In addition, sample preparation remained laborious, as it often involved evaporation, reconstitution, and/or clean-up steps.

To improve the greenness of the analytical procedure, automation, safer solvents and simplified phase-separation strategies have been suggested [29,66]. Hübschen et al. developed an automated VALLME-GC-MS/MS method with injector-based derivatization for the determination of 1,3-dichloropropan-2-ol and 3-chloropropane-1,2-diol in cold-water extracts from food-contact paper and cardboard [74]. The workflow was optimized to minimize solvent consumption, requiring 1.2 mL of ethyl acetate, while enhancing automation by performing derivatization directly in the GC injector, improving the overall method greenness compared with the reference method. Very recently, Cai et al. proposed an amine-switchable hydrophilic-solvent-based homogeneous VALLME for the determination of 2,6-diisopropylaniline in biodegradable poly (butylene adipate-co-terephthalate) (PBAT) films by GC-MS [75]. The novelty of the method was based on the use of amines as switchable hydrophilic solvents, i.e., aqueous solvents capable of reversible switching between homogeneous and biphasic mixtures based on pH switching. The combined use of heating hydrolysis–extraction and switchable hydrophilic extraction provided a green sample-preparation strategy based on the use of a small volume of biodegradable dipropylamine, the elimination of dispersive solvents, and rapid pH-triggered phase separation. Bałdowska et al. developed a VALLME-GC-MS method for the quantitation of eight PAEs in bottled water using eucalyptol as the extraction solvent [76]. The main advantages are the use of a green solvent and direct injection of the extract into the GC-MS system, reducing both the total analytical time and sample handling. Overall, these studies demonstrated that LLME greenness can be enhanced not only by reducing solvent volume but also by selecting safer extractants, integrating procedural steps and improving compatibility with the analytical platform.

In recent years, DESs have received increasing attention as extraction media in LLME because they can be prepared by combining hydrogen-bond acceptors and donors to form liquids with melting points lower than those of the individual components [66,77]. DESs could be considered as greener alternatives to conventional organic solvents due to their tunable polarity, low volatility, low cost and possible use of natural or bio-based constituents. In the analysis of compounds migrating from FCMs, DESs have been proposed, since they form an extractant-rich phase suitable for the preconcentration of hydrophobic migrants, including PAEs, bisphenols, alkylphenols, adipates and aromatic amines. However, the greenness of these approaches should be critically evaluated, since some DES components may be toxic, and the use of disperser solvents or derivatizing reagents may reduce the overall sustainability of the method. In this context, natural hydrophobic DESs (NAHDESs) have gained increasing interest in LLME, as they are composed of bio-based and renewable components, in line with the principles of GAC.

Farajzadeh et al. proposed a hybrid approach using aluminum-fumarate MOF for micro-DSPME in combination with DLLME for the GC-FID analysis of adipate and phthalate esters and butylated hydroxy toluene (BHT) in distillates stored in plastic bottles [78]. This hybrid strategy was designed to enhance analyte enrichment by combining solid-phase extraction with liquid-phase preconcentration, although relatively low recoveries (27–71%) were obtained.

Among DES-based approaches, different studies have focused mainly on the determination of phthalates and adipates in beverages or food-contact plastics. Özgür et al. reported the use of a ChCl:phenol-based DES and THF for the emulsification LLME-GC-MS quantitation of butyl benzyl phthalate and DEHP released from FCMs [79], using MS/MS analysis for confirmatory purposes. Li et al. designed a class of low-viscosity hydrophobic DESs based on quaternary ammonium salts and water-insoluble fatty alcohols or acids for the VALLME of PAEs from plastic FCMs, followed by GC-MS analysis [80]. The development of low-viscosity DESs overcame different limitations of DES-based microextraction, including direct injection within the chromatographic systems and slow mass transfer. Among the developed approaches, the DES made of *n*-hexyl alcohol and tetrabutylammonium bromide was characterized by the best extraction performance, allowing for the analysis of four model PAEs leached in water lixivium from plastic samples. Santana-Mayor et al. developed a VALLME method for the determination of eight PAEs in beverages, using a ChCl:phenol DES as the extraction solvent and tetrahydrofuran (THF) as the emulsifier agent [81]. The UHPLC-UV method was applied to tea, apple-based beverages and pineapple juice, using MS/MS analysis for confirmatory purposes. To improve method greenness, the same research group tested different NAHDESs based on thymol and medium-chain fatty acids for VALLME-UHPLC-MS/MS analysis of PAEs and DEHA from soft drinks [82] and kombucha packaged in plastics or glass [83]. The best results were achieved using a thymol and octanoic acid mixture (2:1 *v*/*v*), which enabled the quantitative extraction of all PAEs and internal standards. Finally, method applicability to different food matrices and simulants was demonstrated. Compared with ChCl:phenol systems, these NAHDESs are more aligned with GAC principles because they are based on bio-derived components and can be applied to different beverage matrices. In addition, method applicability to different food matrices and simulants was demonstrated.

Chen et al. proposed a NADES-based VALLME strategy coupled with DART-HRMS analysis for the quantitative analysis of PAEs in aqueous food samples and aflatoxins in edible oils [84]. The method exploited temperature-mediated phase transitions of NADESs: after extraction, the NADES phase was solidified by cooling, separated from the sample matrix, then melted and vaporized for DART-HRMS analysis. By exploiting reversible temperature-induced phase transitions of NADESs, the method integrated extraction, enrichment, phase separation, and direct ambient HRMS analysis within a single streamlined platform, improving procedural greenness while maintaining the analytical performance.

A second group of DES-based LLME approaches focused on the extraction of bisphenols from FCMs. Li et al. tested different hydrophobic DESs for the extraction of bisphenols from food-contacted plastics by VALLME and HPLC coupled with fluorescence detection (FLD) [85]. Three hydrophobic DESs were prepared by combining trioctylmethylammonium chloride with decanoic acid, ketoprofen or gemfibrozil. Among them, the decanoic acid-based DES exhibited the lowest viscosity while providing comparable extraction capability for the determination of four bisphenols. More recently, Zhang et al. developed an acid-induced DLLME method based on the in situ formation of hydrophobic DESs for BPA and alkylphenols determination in water and beverage samples [86]. This approach is particularly interesting from a green perspective due to the in situ generation of the extract, thereby eliminating the need for pre-synthesized extraction solvents. In this strategy, hydrochloric acid converted fatty acid salts into hydrophobic fatty acids, leading to the formation of insoluble droplets. BPA and alkylphenols were extracted by in situ formation of hydrophobic DESs with the free fatty acids. The extractant phase was solidified by cooling in an ice bath and diluted in methanol before injection in the HPLC-FLD system. A similar approach was developed by Zhu and coworkers for VALLME of phenolic antioxidants via in situ formation of hydrophobic DESs using medium-chain fatty alcohols [87]. In particular, the target antioxidants acted as hydrogen-bond donors, while n-decanol acted as hydrogen-bond acceptors, allowing DES formation and analyte extraction in a single step.

Finally, Zhang et al. developed various terpene-based NAHDESs for the quantitative analysis of aliphatic aldehydes in drinking water and alcoholic beverages [88]. The NAHDESs were prepared using menthol or thymol as hydrogen-bond acceptors and hexafluoroisopropanol as the hydrogen-bond donor. The developed NAHDESs were liquid at room temperature, with a density higher than water, and characterized by relatively low viscosity, allowing the DES-rich phase to settle at the bottom after centrifugation and to be directly analyzed by HPLC-UV without further dilution.

Overall, LLME-based approaches provide fast, simple and miniaturized procedures for the targeted analysis of FCM-related migrants. Conventional DLLME, SALLE-DLLME, VALLME and homogeneous LLME offer efficient enrichment and low detection limits, but their environmental performance depends on solvent selection and auxiliary reagents. DES- and NADES-based methods represent the most important green evolution of LLME, offering tunable extractants that can reduce the use of volatile organic solvents and improve compatibility with microextraction. Nevertheless, DES-based workflows are not automatically green: the toxicity of DES components, the use of disperser solvents such as THF, additional back-extraction or derivatization steps, viscosity-related limitations and instrumental compatibility need to be critically evaluated.

**Table 4:** LLME extraction methods for the analysis of compounds from FCMs.

Ref	Analytes	FCM	Matrix	Extraction Mode	Platform	Solvent/Reagents	LOD	Linear Range	Max RSD (%)	Recovery Rate (%)
[67]	60 compounds	commercial items	food simulant A, B, C, D1	VALLE	GC-HRMS	*n*-hexane	40 ***	LOQ-400 *	12.6	81–120
[68]	BPA	cans, paper boxes and glass jars	food and food simulants	DLLME	HPLC-UV	acetone/ 1-undecanol	0.001 **	0.009–25 **	5.2	76–104
[69]	7 bisphenols	plastic bottles	surface, tap and bottled water	DLLME	UHPLC-MS/MS	acetone/carbon tetrachloride	0.01–0.015 **	0.09–25 **	10.7	83–91
[70]	6 PAEs and DEHA	plastic bottles	herbal-based soft drinks	DLLME	GC-MS/MS	ethanol/*n*-hexane	5.0–13 *	35–135 *	19	82–111
[71]	6 PAEs and DEHA	plastic bottles	66 commercial beers	DLLME	GC-MS/MS	ethanol/*n*-hexane	5.0–13 *	35–135 *	19	82–111
[72]	7 PAEs	disposable cups	hot drinks	VA-UAE-DLLME	GC-MS	*n*-heptane	0.8–15.4 *	LOQ-100 *	6.3	67–101
[73]	BPA and 6 analogues	cans	canned coffee drinks	LLE +DLLME	UHPLC-MS/MS	acetonitrile/dichloromethane	0.01–0.1 **	0.5–50 **	8	70–98
[74]	1,3-dichloropropan-2-ol 3-chloropropane-1,2-diol	paper, cardboard	drinking water	VALLME	GC-MS/MS	ethyl acetate	0.01–0.16 *	1–150 *	7.6	90–102
[75]	2,6-diisopropylaniline	PBAT films	hydrolysis solution	VALLME	GC-MS	dipropylamine, H_2_SO_4_	3.3 **	14.4–7200 **	4.7	92–105
[76]	8 PAEs	plastic bottle	drinking water	VALLME	GC-MS	eucalyptol	90–240 *	500–50,000 *	11	76–131
[78]	adipate/phthalate esters and BHT	plastic bottles	distillates	DSPE–DLLME	GC-FID	aluminum-fumarate MOF, 1,2-dibromoethane 2-propanol	1.80–3.90 *	LOQ-500 *	9.9	27–71
[79]	butyl benzyl phthalate and DEHP	PET bottles, cups and paper cups	drinking water, juice	LLME	GC-MS	THF/ChCl:phenol	12, 5 *	100–5000 *	3.2	95
[80]	4 PAEs	plastic items	water lixivium	VALLME	GC-MS	*n*-hexyl alcohol/tetrabutylammonium bromide	1 *	5–1000 *	5.6	86–103
[81]	8 PAEs	aluminum and glass bottles	tea samples	VALLME	UHPLC-UV	THF/ChCl:phenol	5.1–15.6 *	0.17–5.5 × 10^3^ *	11	84–120
[82]	14 PAEs + DEHA	plastic bottle	soft drinks	VALLME	UHPLC-MS/MS	thymol/octanoic acid	0.025–1.25 ***	LOQ-250 *	19	63–124
[83]	11 PAEs	plastic and glass bottles	kombucha	VALLME	UHPLC-MS, UHPLC-MS/MS	thymol/octanoic acid	0.07–5.45 *	LOQ-62.50	10	67–120
[84]	8 PAEs, aflatoxins	plastic items	food simulant A and edible oil	VALLME	DART-HRMS	L-menthol/1,4-butanediol ChCl: 1,4-butanediol	0.1–0.5 ** (PAEs) 0.4–0.8 ** (afl)	LOQ-20 (PAEs) LOQ-250 (afl)	8.4 (PAEs) 8.2 (afl)	92–114 (PAEs) 90–109 (afl)
[85]	4 bisphenols	plastic items	hot water	VALLME	HPLC-FLD	decanoic acid trioctylmethyl ammonium chloride	0.3–0.5 *	0.3–700 *	6.9	81–109
[86]	BPA and alkylphenols	-	drinking water beverages	DLLME	HPLC-FLD	sodium octanoate	0.03–0.1 *	0.2–400 *	3.9	96–105
[87]	phenolic antioxidants	plastic items	water, food simulant A, B	VALLME	HPLC-UV	*n*-decanol	0.15–0.25 *	LOQ-500 *	3.4	95–104
[88]	aliphatic aldehydes	commercial items	drinking water and alcoholic beverages	DLLME	HPLC-UV	thymol- hexafluoroisopropanol 1:3	0.1–0.5 *	0.2–400 *	9.6	76–119

* ng/mL; ** ng/g; *** LOQ ng/mL.

## 3. Green Instrumental Analysis: SFC and Direct MS Approaches

Besides sample pretreatment, the instrumental platform plays a key role in defining the overall greenness of analytical workflows for FCM characterization. Green analytical strategies aim at reducing solvent consumption, promoting the use of sustainable solvents, preventing waste generation, and minimizing both the analysis time and energy demand of instrumental operation, while preserving sensitivity, selectivity and confidence in compound identification [27,32]. In this context, two complementary directions are particularly relevant: greener chromatographic separations, using SFC, and direct MS-based approaches, including MALDI and AMS.

### 3.1. Supercritical Fluid Chromatography

SFC represents a greener alternative to conventional LC because it relies mainly on CO_2_-rich mobile phases, generally combined with reduced amounts of organic modifiers to tune elution strength and selectivity [27]. In addition, the low viscosity and high diffusivity of supercritical or subcritical CO_2_ can improve mass transfer and reduce analysis time compared with conventional LC, providing fast and efficient separation. Therefore, SFC has progressively expanded toward multiple research fields, including pharmaceutical, environmental, and food analysis [31]. As for FCM analysis, SFC has been proposed for high-throughput targeted analysis of additives and migrants, focusing on plasticizers, phthalates, UV absorbents, photoinitiators, bisphenol derivatives and antioxidants (Table 5).

Qiu et al. developed a rapid UAE-SFC-UV method for the analysis of 14 UV absorbents from plastic FCMs [89]. Despite the rapid 4.5 min chromatographic separation, the overall workflow was limited by time-consuming and solvent-intensive sample preparation, requiring 10 mL of dichloromethane and 5 mL of methanol for UAE. Moreover, additional sample-pretreatment steps, including clean-up and preconcentration, were required prior to SFC-UV analysis, further increasing the overall complexity of the workflow. An improvement of the workflow was proposed by Huang et al., who developed a UAE-SFC-UV method for the simultaneous determination of DEHP and five photoinitiators in plastic FCMs [90] using a single-step UAE procedure based on acetonitrile with reduced solvent consumption. SFC-based methods have been proposed for the detection of plasticizers. He et al. developed a SFC-MS/MS method for the quantitation of 22 alternative plasticizers, including organophosphate esters, alkyl adipates and citrates in plastic films [91]. The analytes were extracted by UAE using a dichloromethane/*n*-hexane mixture, followed by a clean-up stage using silica SPE. Similarly, Ovchinnikov et al. proposed a PLE-SFC-MS/MS method for the rapid determination of 10 PAEs in paper and cardboard products [92], using methanol as extraction solvent. SFC-MS/MS has also been proposed by Yan et al. for the determination of 10 PAEs in water-based adhesives [93]. Sample preparation involved LE with *n*-hexane, followed by DSPE clean-up using spherical carbon-based particles.

Although SFC can be regarded as a green analytical technique, the main drawback of the proposed strategies relies on the sample pretreatment procedures, which require the use of various extraction solvents, including acetonitrile [90], methanol [92], *n*-hexane [93], dichloromethane/methanol [89], and *n*-hexane/dichloromethane [91], thus reducing the overall greenness of the proposed approaches. This highlights how the use of a greener instrumental technique does not necessarily result in a green analytical method when sample preparation requires large amounts of hazardous solvents or involves time-consuming pre-treatment steps.

**Figure 1:**
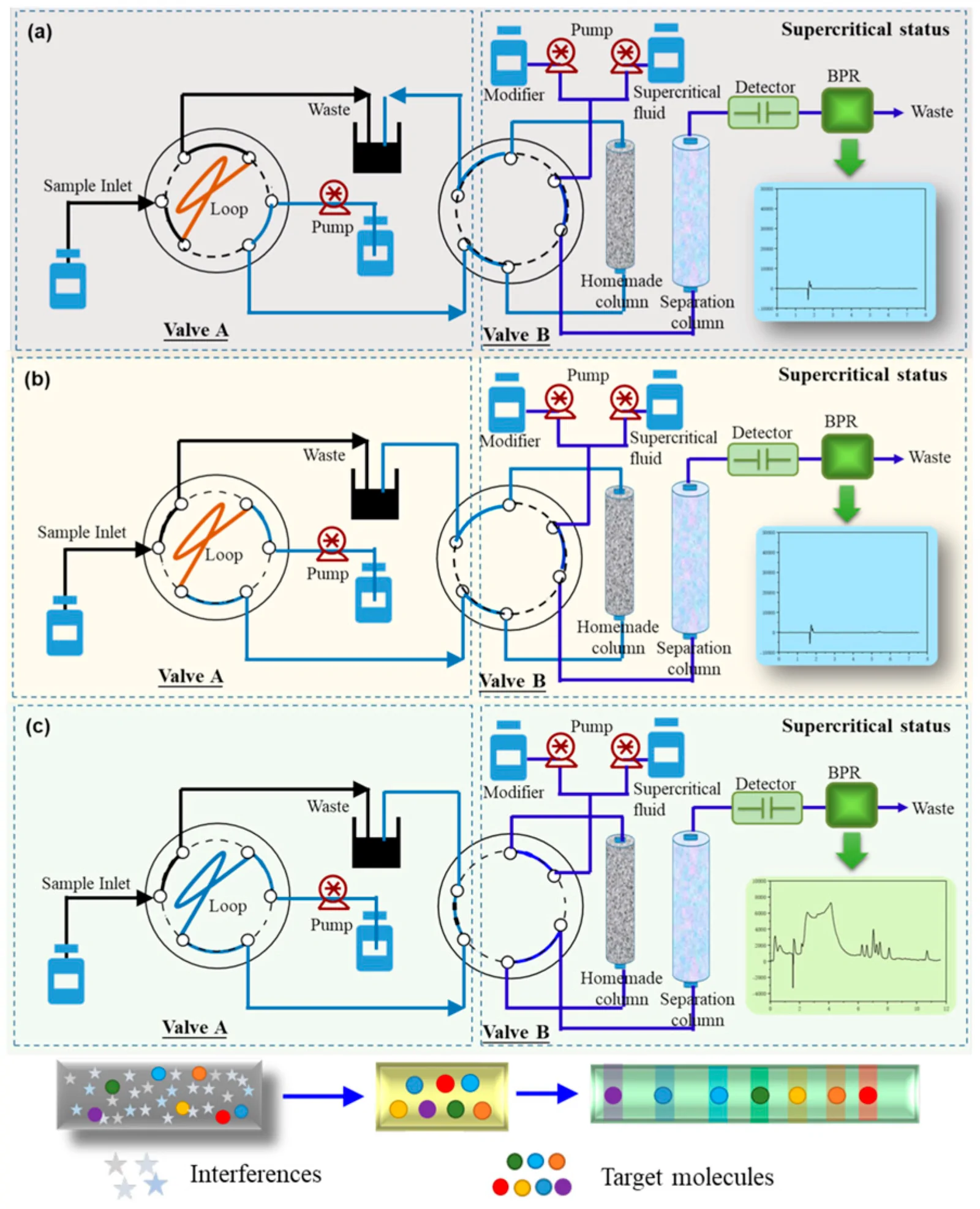
Configuration diagram of the online column-switching SFC system, (**a**) sample loading/system regeneration; (**b**) target molecule enrichment and matrix elimination; and (**c**) analysis and detection of Bisphenol A diglycidyl ethers; reprinted with permission from Lou et al. [95].

To overcome these limitations, more recent applications have integrated SFC with automated or green sample-preparation strategies. Gan and Zhu developed a SFC-UV-based method for the analysis of 12 model compounds belonging to antioxidants, photoinitiators, UV absorbers and plasticizers from edible oils [94]. A modified QuEChERS procedure was applied to speed-up sample pretreatment, allowing for multi-residue analysis. Lou et al. developed an online column-switching SFC method for the determination of bisphenol A diglycidyl ether and its derivatives in canned beverages [95]. In this workflow, a homemade column was used in the first dimension to preconcentrate the analytes and remove matrix interferences online, before SFC separation (Figure 1). This approach is particularly interesting from a GAC perspective because it integrates clean-up, preconcentration and chromatographic analysis into an automated workflow, reducing manual handling and improving high-throughput capability.

**Figure 2:**
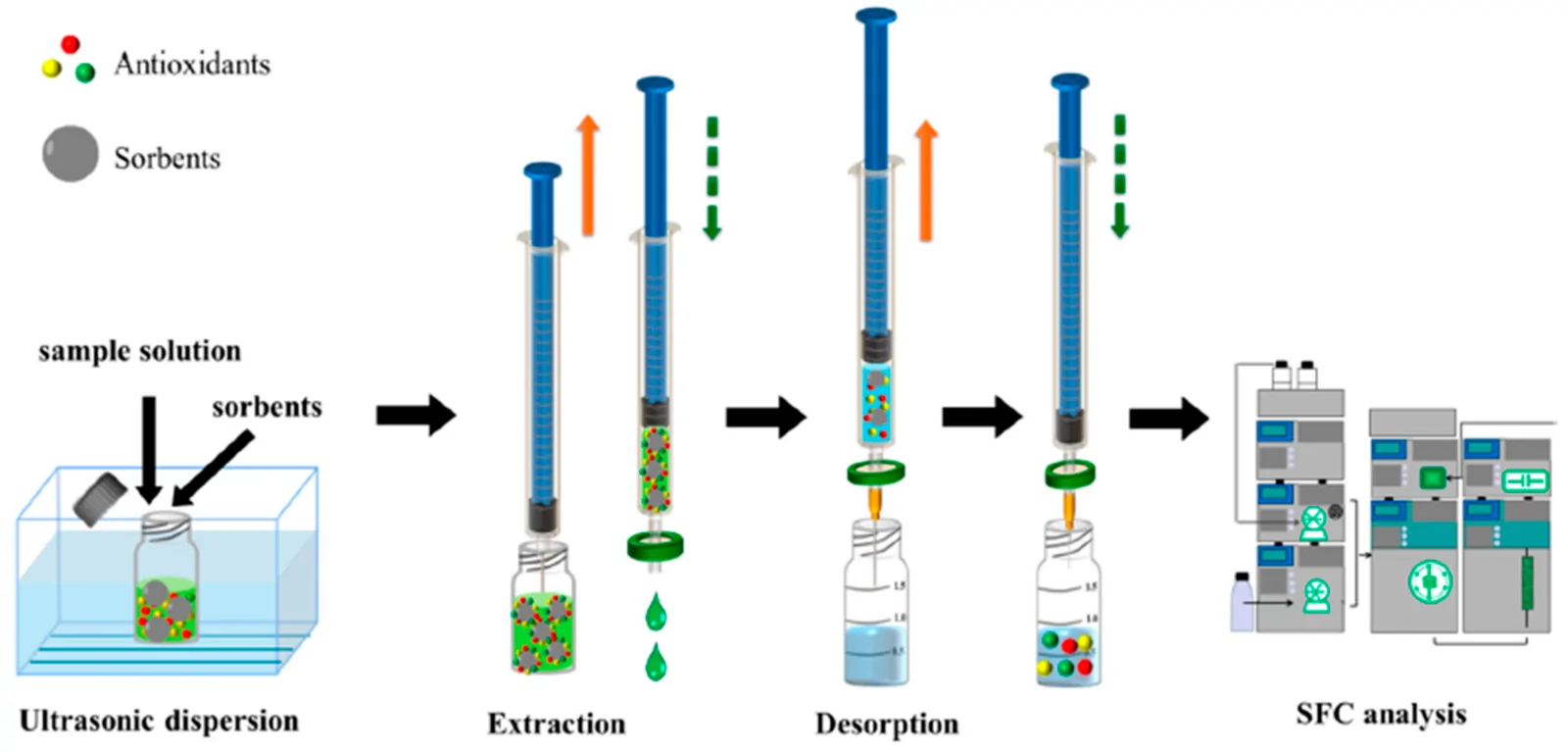
Schematic representation of ultrasonic-assisted d-SPFE-SFC-MS method, reprinted with permission from [96].

A novel UAE-dispersive solid-phase filter extraction (d-SPFE) combined with SFC-UV detection for the determination of six hindered phenolic antioxidants in different food simulants was developed by Pan et al. [96]. The key strength of the proposed UAE-d-SPFE strategy lies in the ultrasonication-assisted dispersion of the sorbent, which enhances sorbent–sample contact and mass transfer, combined with straightforward syringe filtration for rapid sorbent recovery (Figure 2). The analytical workflow allowed complete separation of the six antioxidants within 10 min, highlighting how the integration of green sample pretreatment strategies with SFC-based analysis can achieve high analytical efficiency while improving the overall sustainability of the procedure.

Overall, these studies demonstrate that SFC is emerging as a rapid and greener platform for the targeted analysis of FCM-related compounds. The main advantages include reduced organic solvent consumption, short chromatographic run times, and compatibility with multiple detection techniques. However, current applications remain limited to targeted analysis. As previously stated, the overall greenness of a method depends not only on the separation technique but also on the sample preparation procedure, which often relies on conventional solvent-based extraction and laborious procedures. In this context, the integration of green sample pretreatment procedures with SFC is fundamental for the development of reliable, high-throughput analytical workflows for both the targeted and untargeted determination of IAS and NIAS from FCMs.

### 3.2. Direct Mass Spectrometry Approaches for the Analysis of FCMs

Direct MS approaches represent complementary techniques for the analysis of FCMs, bypassing chromatographic separation and minimizing sample treatment [22,23]. MALDI and AMS techniques can provide rapid chemical fingerprints of materials, extracts or food simulants, supporting high-throughput screening, suspect prioritization and the preliminary identification of additives, oligomers, degradation products and other IAS/NIAS (Table 6). Ambient MS is particularly aligned with GAC principles, since it enables direct analysis, reduces or avoids the use of solvents and reagents, and integrates sampling, desorption/ionization and detection into a single step.

MALDI-MS has been frequently proposed for the analysis of polymeric and oligomeric species. Its main advantage is the possibility of obtaining rapid molecular fingerprints and molecular-weight distributions of polymer-related compounds, without the need for chromatographic separation. Lawson et al. applied MALDI-MS to investigate migrants from polyurethane adhesives used in multilayer food packaging laminates [97]. The method was used to obtain fingerprint patterns of polyether and polyester polyol components and to identify migrating species into water. The study confirmed the migration of unreacted polyol components from polyether-based adhesive systems, whereas cyclic oligomers were identified as migrants from polyester-based adhesives. However, this study also highlighted a major limitation of MALDI-MS, i.e., its limited quantitative capability, thus requiring complementary analytical techniques when accurate quantification is needed. Colombo et al. combined MALDI-MS with UHPLC-HRMS for the untargeted screening of NIAS and cyclic oligomers migrating from virgin and recycled PET food trays in simulant D1 [98]. MALDI-MS proved to be an effective tool for evaluating the molecular weight distribution of PET trays, revealing a decrease in molecular weight distribution in recycled PET compared with virgin PET. Conversely, UHPLC-HRMS was more suitable for investigating the migration of IAS, NIAS and cyclic oligomers into the food simulant. The study showed that cyclic oligomer migration depended on both the virgin/recycled PET ratio and the extrusion technology, identifying process-specific markers. Zhang et al. employed MALDI-HRMS, together with gel permeation chromatography and UHPLC-HRMS, for the comprehensive characterization of oligomers in the biodegradable polyesters poly (butylene succinate) (PBS) and PBAT [99]. Cyclic esters with repeating units from 2 to 8 were detected in PBS, whereas PBAT showed cyclic esters with repeating units from 1 to 10, including random combinations derived from its different monomers. The combination of complementary platforms provided both global molecular-weight information and detailed structural elucidation, allowing a more comprehensive assessment of oligomer distributions. As a general comment, it can be stated that although the use of complementary analytical platforms substantially enhances the characterization of migrants and oligomers, their overall greenness should be carefully considered. Although MALDI-MS provides a relatively low-solvent and rapid screening approach, the additional use of chromatographic and HRMS techniques increases solvent consumption, energy demand, and workflow complexity.

AMS has been applied as a rapid screening tool for FCMs, particularly for high-throughput screening or preliminary investigation. Paseiro-Cerrato et al. applied DART-HRMS as a screening technique, followed by GC-MS confirmation, to identify brominated flame retardants (BFRs) in reusable food contact articles [100]. Several BFRs were detected within the investigated samples, with estimated false-positive and false-negative rates of 5% and 4%, respectively. A related comprehensive strategy was reported by Lestido-Cardama et al., who combined DART-HRMS, XRF and HPLC-MS/MS for the determination of BFRs in food contact articles [101]. DART-HRMS and XRF were used as screening tools, while a validated HPLC-MS/MS was used for the quantification of tribromophenol, tetrabromobisphenol-A, and decabromodiphenyl ether. In particular, DART-HRMS demonstrated better detection capabilities compared to XFS, showing false positive rates and incidences of 6% and 5%, respectively. The positive samples were then subjected to migration testing using simulant C and D2: HPLC-MS analysis demonstrated the migration of some BFRs, with levels up to 90 μg kg^−1^.

DART-HRMS has also been applied to PFAS screening in FCMs. Scholl et al. developed a DART isotope dilution (DART-ID)-HRMS method for the detection of 6:2 fluorotelomeric alcohol (6:2-FTOH) in hydrolyzed food contact paper [102]. The workflow was based on the saponification of ester-linked fluorotelomer side chains, followed by rapid semi-quantitative detection of 6:2-FTOH in the headspace without preconcentration or chromatography. ^19^F-NMR was used for confirmatory purposes. By using labelled 6:2-FTOH for light/heavy ion-ratio correction, the DART-HRMS method achieved estimated LODs of 2.87 ng/g in hydrolysis solution and 11.7 ng/g in FCP, supporting its use for the rapid monitoring of ester-linked fluorotelomer grease-proofers. Ackerman et al. further validated a hydrolysis DART-ID-HRMS method for the regulatory screening of 6:2-FTOH-containing grease-proofers in fiber-based food packaging [103]. The method identified hydrolyzable 6:2-FTOH across different packaging types, with no false positives or false negatives and a linear response in the 4 μg/kg–130 mg/kg range for hydrolyzed 6:2-FTOH. In addition, the analysis required approximately 14 min per sample, showing its suitability for monitoring purposes.

Meng et al. developed a DART-HRMS method for the rapid analysis of four pyrethroid preservatives in wooden FCMs [104]. The sawdust of wooden FCMs was extracted using *n*-hexane/acetone (1:1, *v*/*v*), followed by DSPE using 5 mg of C18 silica particles and 10 mg of primary secondary amine for extract clean-up. The supernatant was dried, re-dissolved in acetonitrile and loaded on the metal mesh for DART-HRMS analysis. Validation provided LODs in the 0.04–0.20 mg/kg range, recoveries between 72.1% and 82.7%, and RSD < 12%.

AMS has also been investigated by Osorio et al. for assessing the migration of FCCs from bio-based and biodegradable packaging [106,107]. In the first study, migrants from a bamboo-based biopolymer were analyzed by GC-MS for the determination of volatile and semi-volatile compounds, UHPLC-HRMS was applied for the detection of non-volatile compounds, while DART-MS was evaluated for screening purposes [105]. GC-MS detected 6, 15 and 6 compounds in food simulant A, B, and D2, respectively, for a total of 25 compounds, whereas UHPLC-HRMS allowed the detection of 12 non-volatile migrants, mainly melamine and related derivatives. Finally, DART-MS was able to rapidly detect 26 migrating compounds, showing its potential as a fast tool for safety and compliance testing of packaging materials. In the second study, UHPLC-HRMS, DART-MS and ASAP-MS were compared for the analysis of oligomers migrating from PLA and starch-based FCMs into food simulants [106]. UHPLC-HRMS detected 14 cyclic and 5 linear oligomers from PLA samples, whereas 19 polyester, 12 cyclic, and 2 linear oligomers were observed in starch-based samples, possibly deriving from PBAT. DART analysis enabled the detection of polyester oligomers over the *m/z* 50–1000 range within 1.5 min per replicate sample. By contrast, ASAP-QTOF-MS mainly detected lower-molecular-weight polyester oligomers, suggesting its use for the rapid screening of volatile and semi-volatile oligomeric species.

A different green workflow was proposed by Duenas Mas et al., combining SUPRAS extraction with AMS for the determination of organic contaminants in FCMs [107]. SUPRASs made of medium-chain alcohol in ethanol/water mixtures were used to extract contaminants from FCMs, providing simultaneous extraction and clean-up. The optimized procedure used 20 mg of FCM sample and 400 µL of 1-decanol-based SUPRAS. The SUPRAS-ASAP-MS/MS method was applied for the quantitative determination of selected bisphenols and aryl-organophosphate flame retardants, obtaining LODs in the 0.01–0.4 µg/g. In addition, the use of a direct injection probe was tested for untargeted purposes. The developed workflow enabled the tentative identification of 14 additional compounds in positive samples, including plastic additives such as UV filters, fatty acids, phthalates or synthesis intermediates, as well as compounds likely originating from food residues or insufficient cleaning of the packaging.

Overall, MALDI-MS and AMS offer complementary advantages for FCM analysis, providing high-throughput platforms for screening purposes. However, both techniques present important limitations: MALDI ionization is strongly sample-dependent, and quantitative analysis may be challenging due to ionization variability. AMS methods can suffer from matrix effects, ion suppression, isobaric interferences and lower identification confidence when chromatographic separation is not used. Therefore, these techniques should be regarded as complementary tools for rapid screening, fingerprinting, suspect prioritization and polymer/oligomer characterization, requiring the use of chromatographic-based techniques for confirmatory purposes. Future developments should aim to retain the complementary information provided by these techniques while reducing redundant analytical steps and resource consumption.

## 4. Greenness Assessment Metrics

An objective evaluation of analytical greenness requires the use of dedicated assessment metrics. Over the last two decades, several tools have been developed to traduce the GAC principles into quantitative or semi-quantitative scores as well as pictograms or color-coded outputs, considering parameters such as reagent toxicity, solvent volumes, waste generation, energy consumption, operator safety, extent of sample preparation, analytical throughput, and sustainability of solvents and reagents [108,109]. More recently, the evaluation of analytical methods has expanded beyond simple greenness, moving toward the concept of White Analytical Chemistry (WAC), where a balance between the environmental impact, analytical performance and practical applicability is considered. In this context, green metrics assess environmental and safety aspects, blue metrics evaluate applicability, throughput and cost, and red metrics focus on analytical performance. To consider such a complex view, multidimensional tools integrate the different dimensions into broader sustainability assessments. Several reviews have been recently published that focus on the different metrics and their applicability [108,109,110].

Among the most widely used tools, the Analytical GREEnness Metric Approach (AGREE) evaluates the greenness of the whole analytical procedure [111]. The output includes both a numerical score, ranging from 0 to 1, with 1 indicating full compliance with green principles, and a radial visual representation divided into 12 sections, each corresponding to one GAC principle. A modified tool, AGREEprep, specifically focuses on the sample-preparation step and is based on the 10 principles of green sample preparation, including in situ preparation, the use of safer solvents and reagents, renewable or reusable materials, waste minimization, reduced sample and chemical consumption, increased throughput, integration and automation, lower energy consumption, compatibility with greener analytical techniques, and operator safety [112].

Another widely used family of greenness metrics is based on the Green Analytical Procedure Index (GAPI) and its modifications, including MoGAPI, ComplexGAPI and ComplexMoGAPI. GAPI provides a semi-quantitative graphical assessment of the analytical workflow through a color-coded pictogram, allowing rapid visualization of critical steps related to sample collection, preservation, transport, preparation, reagents and solvents, instrumentation, waste generation and waste treatment [113]. In contrast to AGREE, which provides a single numerical score, GAPI is particularly useful for identifying which steps of the analytical procedure have the greatest environmental impact. Modified versions of GAPI have been developed to address some limitations of the original approach, providing a quantitative interpretation of the results and expanding the assessment to more complex workflows. In particular, ComplexGAPI is well suited to methods involving pre-analytical steps or additional operations, such as the synthesis of extraction materials, clean-up procedures or auxiliary sample-treatment steps; thus, these need to be considered.

The Blue Applicability Grade Index (BAGI) was introduced to provide both a numerical and visual evaluation of the analytical method based on WAC [114]. In this context, type of analysis, number of analytes, instrumentation, sample throughput, sample preparation, reagents and materials, automation and sample amount are considered. Additional tools, such as the RGB model, RGB12 and RGBfast, have been developed to provide a more balanced assessment of analytical methods by simultaneously considering the red, green and blue dimensions, thereby extending the evaluation beyond greenness alone.

The selection of a metric should be carefully evaluated, since each tool addresses different aspects of the analytical workflow and provides distinct and often complementary information. A major concern associated with greenness metrics is the risk of drawing misleading conclusions when inappropriate or inconsistent evaluation criteria are applied, especially when comparing methods that differ substantially in scope, sample preparation, instrumentation or analytical purpose. In fact, no single metric can comprehensively capture all aspects of analytical sustainability and performance, and the outputs may vary substantially among tools. Therefore, greenness scores should not be interpreted as absolute indicators of method quality but rather as complementary tools to support transparent comparison among analytical workflows. This is particularly important in FCM analysis, where method greenness should be evaluated together with analytical performance and practical applicability, ensuring adequate sensitivity, selectivity, robustness and identification confidence.

Despite the increasing availability of greenness metrics, their routine application in FCM studies remains limited. Most published methods do not report standardized AGREE, AGREEprep, GAPI or related scores. This represents a current gap in the field and limits direct comparison between conventional and green workflows. Future studies should therefore include metric-based greenness assessment together with conventional validation parameters, allowing a more balanced evaluation of sustainability, applicability and analytical reliability.

It should also be considered that targeted and non-targeted workflows cannot be evaluated using the same criteria. In fact, current greenness and whiteness metrics are well suited to targeted methods, whereas their application for the evaluation of non-targeted workflows is very limited. In this context, additional aspects become critical, including chemical coverage, feature detectability and reproducibility, data analysis, database availability, spectral-library coverage, annotation confidence, and false positive and false negative annotations. Conversely, a non-targeted workflow may require higher energy consumption, extensive data processing and confirmatory analyses while providing broader chemical information than a target method. Therefore, future assessments should be aimed at combining conventional GAC/GSP or WAC metrics with additional non-target-specific quality indicators.

## 5. Conclusions and Perspectives

The studies discussed in this review show a clear transition toward greener, faster and more integrated workflows for the characterization of IAS and NIAS in FCMs. The development and application of green-sample preparation strategies have reduced solvent consumption, sample handling and extraction time. In this context, the development of new protocols based on miniaturized sample pretreatment techniques and the synthesis of novel sorbent materials play a pivotal role, reducing both sample and solvent volumes while increasing the sensitivity and selectivity of the analytical workflow. At the same time, greener instrumental platforms such as SFC, MALDI-MS and AMS enable faster separations, rapid material screening, and high-throughput prioritization of contaminated or critical samples.

Despite these advances, current applications remain largely targeted and class-specific, focusing mainly on plasticizers, bisphenols, antioxidants, and other IAS. Comprehensive untargeted workflows capable of covering the broad chemical diversity of IAS and NIAS are still limited. In addition, the greenness of a method should consider both sample pretreatment and analytical determination, while current workflows are mainly focused on the improvement of single analytical steps. Future developments should focus on combining green extraction with HRMS-based suspect and non-target screening, improving spectral libraries and identification confidence, validating rapid direct-MS approaches, and integrating chemometrics for material comparison and sample prioritization. Attention should also be given to recycled, bio-based and multilayer materials, where chemical complexity, degradation products and unknown NIAS pose significant analytical challenges. In this perspective, the rational integration of green sample preparation and analysis will be essential for developing sustainable, reliable highly informative workflows for FCM safety assessment.

## Figures and Tables

**Table 1:** SPME-based methods for the analysis of compounds from FCMs.

Ref	Analytes	FCM	Matrix	Extraction Mode	Platform	Coating	LOD	Linear Range	Max RSD (%)	Recovery Rate (%)
[40]	6 PAEs	plastic bottles	drinking water	DI	GC-MS/MS	50/30 μm DVB/CAR/PDMS	0.3–2.6 *	5–500 *	5.8	89–103
[41]	DMP, DEP, DPP	plasticizer-free FCM	food simulant A, B, C, D, E	DI	GC-MS/MS	65 µm DVB/PDMS	29–73 *	LOQ-10,000 *	26.9	88–98
[42]	volatile silicone oligomers and migrants	silicone molds	Tenax^®^ as food simulant	HS	GC-MS	PDMS	0.09–0.18 **	LOQ-130 **	65.8	-
[43]	MOAH	hot-melt adhesives	Tenax^®^ as food simulant	HS	GC-MS	100 μm PDMS	12–113 **	0.5–31.6 × 10^3^ **	10.1	83–106
[44]	untargeted	plastic multilayer and cartons	food simulant D1	DI	GC-MS	65 μm PDMS/DVB	0.25–14.3 *	5–100 1–10 × 10^3^ *	21.6	-
[45]	6 PAEs	plastic bottles	soft drinks	DI	GC-MS	biopolymer-ionic liquid-modified clay bionanocomposite	0.001 **	0.01–400 **	5.8	89–109
[46]	8 PAEs	plastic bottles	drinking water	DI	GC-MS	tetraphenylethylene-1,4- bis (chloromethyl) benzene hypercrosslinked polymer	2–22 × 10^−3^ *	LOQ-10 *	9.3	75–116%
[47]	9 PAEs	plastic bottles	liquid food samples	DI	GC-MS	quinoxaline-based conjugated microporous polymer	0.014–0.032 *	0.5–50 *	6.6	70–96
[48]	14 PAEs	plastic bottles	drinking water	DI	UHPLC-MS/MS	CIM-80 (Al)	0.01–0.05 *	LOQ-1000 *	18.8	70–107
[49]	5 PAEs	plastic bottles	tea	DI	GC-FID	1,3,5-tris (aminophenyl) benzene- 2,5-dimethoxy terephthalaldehyde COF	40–440 *	40–500,000 *	14.1	85–115
[50]	aromatic amines	PE bags	food simulant B and C	DI	GC-MS/MS	carbon nanotube cage	0.1–2 × 10^−3^ *	0.5–500 × 10^−3^ *	8.6	82–118
[51]	vinyl acetate monomer	ethylene vinyl acetate copolymer	food simulant A, distilled water and saline solution	HS	GC-MS	ZIF-68	0.1 *	0.001–10 × 10^−3^ *	7.6	85%

* ng/mL; ** ng/g.

**Table 2:** Untargeted SPME-based methods for the analysis of compounds from FCMs.

Ref	Analyte	FCM	Matrix	Extraction Mode	Platform	Coating	Number of Annotated Features
[52]	volatile and semi-volatile IAS/NIAS	recycled polyolefins	simulants A, B and D2 substitute	DI	GC-MS	50/30 μm DVB/CAR/PDMS	35
[53]	off-odors	recycled polypropylene	direct analysis	HS	GC-O-MS	50/30 μm DVB/CAR/PDMS	45
[54]	off-odors	recycled PLA	direct analysis	HS	GC-O-MS	50/30 μm DVB/CAR/PDMS	34
[55]	volatile nonvolatile IAS/NIAS	starch-based FCMs	simulants A, B and D2 substitute	HS	GC-MS, UHPLC-HRMS	50/30 μm DVB/CAR/PDMS	15 volatile 11 nonvolatile
[56]	volatile nonvolatile IAS/NIAS	vacuum cooking bags	simulants A, B and D2 substitute	DI	GC-MS, UHPLC-IMS-HRMS	50/30 μm DVB/CAR/PDMS	64 volatile 29 nonvolatile
[57]	volatile IAS/NIAS	recycled PET and polyolefins	food simulants A, B	HS	GC-MS	50/30 μm DVB/CAR/PDMS	121 in PET 178 in polyolefins
[16]	volatile IAS/NIAS	cosmetic plastic packs	direct analysis	pyrolysis	GC-MS	100 μm PDMS	35

**Table 3:** DSPE and MSPE methods for the analysis of compounds from FCMs.

Ref	Analytes	FCM	Matrix	Extraction Mode	Platform	Material	LOD	Linear Range (ng/mL)	Max RSD (%)	Recovery Rate (%)
[59]	BPA and bisphenol AF	disposable plastics	hot water, food simulant A, B, C, D1	DSPE	UHPLC-HRMS	ZnO@CTAB	0.027–0.030 *	0.1–100	3.5	98–109
[21]	antioxidants and UV stabilizers	PLA	dissolution-precipitation	MSPE	UHPLC-MS/MS	MIL-101(Fe)/SA	0.023–3.105 **	LOQ-40,000	9.8	71–102
[60]	phenolic antioxidants	PE	milk	DSPE	HPLC-UV	Carbon-derived MIL-88(Fe)	0.19–0.63 **	LOQ-100	5.8	82–111
[61]	alternative plasticizers	HDPE packaging	protein supplements and acetonitrile	UAE, DSPE/SPE clean-up	GC-MS/MS	SiO_2_-PSA	0.75–288 × 10^−3^ ***	5–400	15	80–96
[62]	110 chemicals from printing inks and adhesives	commercial items	acetonitrile	QuEChERS+ DSPE clean-up	GC-MS/MS LC-MS/MS	C_18_/PSA	0.2–120 ***	10–500	22	60–140
[63]	6 PAEs	plastic bottles	drinking water	MSPE	UHPLC-UV	carbon shell-decorated magnetic microspheres	0.08–0.3 *	LOQ-100	8.1	88–104
[64]	4 PAEs	plastic bottles	fruit juice	DSPE	GC-FID	NiCoMn-MOF	0.11–0.29 *	LOQ-250	6.8	65–75

* ng/mL; ** ng/g; *** LOQ ng/g.

**Table 5:** SFC-based methods for the analysis of compounds from FCMs.

Ref	Analytes	FCM	Matrix	Extraction Mode	Platform	Extractant	LOD	Linear Range	Max RSD (%)	Recovery Rate (%)
[89]	14 UV absorbents	PET items	-	UAE	SFC-UV	dichlorometane/ methanol	30–100 *	0.1–50 × 10^3^ **	7.2	90–114
[90]	DEHP, 5 photoinitiators	plastic items	-	UAE-SPE	SFC-UV	acetonitrile	21–150 *	LOQ-20 × 10^3^ *	8.1	91.2–108.3
[91]	22 alternative plasticizers	plastic films	-	UAE-SPE	SFC-MS/MS	dichloromethane/*n*-hexane	0.04–10 **	LOQ-500 **	10.9	76–124
[92]	10 PAEs	paper and cardboard products	-	PLE	SFC-MS/MS	methanol	0.02–0.28 **	2–400 **	10	74–135
[93]	10 PAEs	water-based adhesives	-	LE+DSPE	SFC-MS/MS	*n*-hexane and spherical carbon-based particles	15–29 **	10–500 *	7	83–100
[94]	12 model antioxidants, photoinitiators, UV absorbers and plasticizers	commercial items	edible oils	QuEChERS	SFC-UV	acetonitrile	50–150 *	0.2–20 × 10^3^ *	10	61–106
[95]	BPA diglycidyl ether and derivatives	cans	soft drinks	online extraction	SFC-UV	water/ methanol	2.4–3.5 *	0.2–10 × 10^3^ *	12	86–106
[96]	6 hindered phenolic antioxidants	commercial items	pure water, food simulant B, C	UAE-d-SPFE	SFC-UV	EVB/DVB microspheres, acetonitrile	2.4–3.6 *	0.2–20 × 10^3^ *	10	89–102

* ng/mL; ** ng/g.

**Table 6:** Direct MS-based methods for the analysis of compounds from FCMs.

Ref	Analytes	FCM	Extraction Mode	Matrix	Platform
[97]	untargeted	multilayer laminates	direct analysis	-	MALDI-MS
[98]	IAS, NIAS and cycle oligomers	virgin and recycled PET trays	migration	food simulant D1	MALDI-MS UHPLC-HRMS
[99]	oligomers	PBS and PBAT samples	LE	organic solvent mixtures	MALDI-HRMS
[100]	BFRs	reusable articles	direct analysis	-	DART-HRMS
[101]	BFRs	commercial items	direct analysis/migration	food simulant C, D2 substitute	DART-HRMS, XRF, HPLC-MS/MS
[102]	6:2-FTOH	food contact paper	hydrolysis+saponification	HS	DART-ID-HRMS
[103]	6:2-FTOH	fiber-based food packaging	hydrolysis	HS	DART-ID-HRMS
[104]	4 pyrethroid preservatives	wooden items	LE+DSPE	*n*-hexane/acetone	DART-HRMS
[105]	untargeted	bamboo-based biopolymer	migration	food simulant A, B, D2 substitute	GC-MS, UHPLC-HRMS, DART-MS
[106]	oligomers	PLA and starch-based items	migration	food simulant A, B, D2 substitute	UHPLC-HRMS, DART-MS, ASAP-MS
[107]	bisphenols and aryl-organophosphate flame retardants, untargeted	commercial items	LE extraction	1-decanol SUPRAS	ASAP-MS

## Data Availability

No new data were created or analyzed in this study. Data sharing is not applicable to this article.

## Abbreviations

6:2-FTOH	6:2 fluorotelomeric alcohol
AMS	ambient mass spectrometry
ASAP	atmospheric solids analysis probe
BFR	brominated flame retardant
BHT	butylated hydroxy toluene
BPA	bisphenol A
CAR	carboxen
ChCl	choline chloride
CPME	cyclopentyl methyl ether
d-SPFE	dispersive solid-phase filter extraction
DART	direct analysis in real time
DART-ID	direct analysis in real time isotope dilution
DEHA	bis(2-ethylhexyl) adipate
DEHP	di-ethylhexyl phthalate
DEP	diethyl phthalate
DES	deep eutectic solvent
DHS	dynamic headspace
DI	direct immersion
DLLME	dispersive liquid–liquid microextraction
DMP	dimethyl phthalate
DPP	dipentyl phthalate
DSPE	dispersive solid-phase extraction
DVB	polydivinylbenzene
DVS	dynamic vapor sorption
EF	enrichment factor
FCC	food contact chemical
FCM	food contact materials
GAC	green analytical chemistry
GC-FID	gas chromatography-flame ionization detection
GC-MS	gas chromatography-mass spectrometry
GC-O-MS	gas chromatography-olfactometry-mass spectrometry
HDPE	high-density polyethylene
HPLC-FLD	high-performance liquid chromatography-fluorescence detection
HRMS	high-resolution mass spectrometry
HS	headspace
IAS	intentionally added substances
IL	ionic liquid
IMS	ion mobility spectrometry
IT	in-tube
LC-MS	liquid chromatography-mass spectrometry
LC-UV	liquid chromatography-UV detection
LOD	limit of detection
LOQ	limit of quantitation
MAE	microwave-assisted extraction
MALDI	matrix-assisted laser desorption/ionization
MOF	metal organic framework
MOHA	mineral oil aromatic hydrocarbon
MS/MS	tandem mass spectrometry
MSPE	magnetic solid phase extraction
NAHDES	natural hydrophobic deep eutectic solvent
NIAS	non-intentionally added substances
P&T	purge-and-trap
PAE	phthalic acid ester
PBAT	poly(butylene adipate-co-terephthalate)
PBS	poly(butylene succinate)
PDMS	polydimethylsiloxane
PE	polyethylene
PET	polyethylene terephthalate
PLA	poly(lactic) acid
PLE	pressurized liquid extraction
PP	polypropylene
QuEChERS	Quick, Easy, Cheap, Effective, Rugged and Safe
RSD	relative standard deviation
SFC	supercritical fluid chromatography
SPE	solid-phase extraction
SPME	solid-phase microextraction
SUPRAS	supramolecular solvents
THF	tetrahydrofuran
UAE	ultrasound-assisted extraction
UHPLC	ultra-high-performance liquid chromatography
VA	vortex-assisted extraction
WAC	white analytical chemistry
ZnO@CTAB	cetyltrimethylammonium bromide-intercalated zinc oxide
